# Emergence of *Babesia canis* in southern England

**DOI:** 10.1186/s13071-017-2178-5

**Published:** 2017-05-17

**Authors:** Maria del Mar Fernández de Marco, Luis M. Hernández-Triana, L. Paul Phipps, Kayleigh Hansford, E. Sian Mitchell, Ben Cull, Clive S. Swainsbury, Anthony R. Fooks, Jolyon M. Medlock, Nicholas Johnson

**Affiliations:** 1 Wildlife Zoonoses and Vector-Borne Diseases Research Group, Animal and Plant Health Agency, Woodham Lane, Addlestone, Surrey KT15 3NB UK; 2 Medical Entomology and Zoonoses Ecology, Emergency Response Department, Public Health England, Porton Down, Salisbury, SP4 0JG UK; 3 Animal and Plant Health Agency Carmarthen, Jobs Well Road, Johnstown, Carmarthen SA31 3EZ UK; 4 Forest Veterinary Centre, Eastwick Lodge, Harlow, Essex CM20 2QT UK; 5 Department of Clinical Infection, Microbiology and Immunology, Institute of Infection and Global Health, University of Liverpool, Liverpool, UK; 6 NIHR Health Protection Research Unit in Emerging and Zoonotic infections, Salisbury, UK; 7 Faculty of Health and Medical Science, University of Surrey, Guildford, Surrey GU2 XH UK

**Keywords:** *Babesia canis*, *Dermacentor reticulatus*, Canine babesiosis

## Abstract

**Background:**

The United Kingdom is considered free of autochthonous transmission of canine babesiosis although cases are reported in dogs associated with recent travel abroad. During the winter months of 2015/16, a cluster of cases of disease in dogs with signs suggestive of canine babesiosis were reported in Harlow, Essex.

**Methods:**

*Babesia* species were detected in dog blood samples by Giemsa staining of blood smears and by pan-piroplasm PCRs. *Babesia* species were also detected in extracts of tick DNA using pan-piroplasm PCRs. DNA sequencing and phylogenetic analysis was used to confirm the species of *Babesia* present in dog blood and tick samples. Tick species were identified by PCR-sequencing based on amplification of the cytochrome *c* oxidase subunit one (*cox*1) gene. *Dermacentor reticulatus* ticks were sampled from field sites in England and Wales.

**Results:**

Blood smear analysis on samples taken from some of the affected dogs confirmed the presence of a large *Babesia* species within erythrocytes. A tick recovered from one of these cases was identified as *Dermacentor reticulatus*, a species with a limited distribution in England and Wales, but a known vector of canine babesiosis in continental Europe. *Babesia canis* was subsequently identified in blood samples obtained from three clinical cases (all dogs) within the area and from ticks associated with these dogs. A field survey detected 17 adult *D. reticulatus* ticks from one area visited by the affected dogs. Fourteen of these ticks were shown to be positive for the *B. canis* parasite, implicating them as a potential source for babesiosis in Harlow. In order to assess whether the parasite is present in more than one tick population, *D. reticulatus* ticks from across England and Wales were screened for the presence of *Babesia* species. In addition to the Harlow site, a further five locations where *D. reticulatus* is present were screened for *Babesia* species. *Babesia* was not detected from most sites tested but one tick from a single location in Wales was positive for *B. canis*.

**Conclusions:**

Infection with *B. canis* was confirmed in a number of dogs in Harlow, Essex, with no history of travel outside of the country. The same pathogen was identified in field-caught *D. reticulatus* ticks in the same area and is considered the likely source of infection. This highlights the need for vigilance by veterinary surgeons for future outbreaks of tick-borne disease in dogs.

## Background

Babesiosis is one of the major tick-borne infections of dogs around the world [1]. It is caused by haemoprotozoan parasites, or piroplasms, belonging to the genus *Babesia* that infect erythrocytes in the vertebrate host. In extreme cases, the dog exhibits fever, jaundice, and anaemia that if left untreated can lead to death. However, the disease is treatable if recognised [1], although there are no treatments currently licensed for use in dogs in the UK. The underlying disease mechanisms are poorly understood and can present as a range of disease signs that if not treated develop into a number of immune-mediated syndromes [2]. One potential cause for variation in disease severity is the species of *Babesia*. In Europe, four species of piroplasm have been reported to cause clinical disease in dogs including *B. canis*, *B. vogeli*, *B. gibsoni* and *B. microti*-like [3]. In addition, “*Theileria annae*” has been reported to cause severe regenerative anaemia, renal failure and thrombocytopenia in dogs [4]. This species has tentatively been renamed “*Babesia vulpes* sp. nov.” [5], although this is disputed. The most commonly encountered species are *B. canis*, associated with transmission by *Dermacentor reticulatus* [6] and *B. vogeli* associated with transmission by the brown dog tick, *Rhipicephalus sanguineus* [7]. Although other piroplasms including *B. caballi* [8] and *Theileria equi* [9] have been detected in the blood of dogs in Europe using molecular methods; their significance in the epidemiology of disease is unknown at this time.

Canine babesiosis caused by *B. canis* is highly prevalent in countries of southern and central Europe including France [10], Portugal [11] and Hungary [12]. The disease is rarely encountered in the UK and is usually associated with dogs returning to the UK after travel abroad to countries where the disease is endemic [13–15]. However, occasional cases have been reported where autochthonous transmission has been suspected [16]. The absence of canine babesiosis was believed in part due to the absence of key tick vectors such as *R. sanguineus* and the restricted distribution of *D. reticulatus*, being limited to coastal locations in Devon, West Wales and Essex [17, 18] or to poor reporting of clinical cases. In recent years, there has been a significant increase in dogs travelling with owners from the UK to continental Europe as a result of the introduction and modification of the Pet travel scheme (PeTS) [19, 20], enabling dogs vaccinated against rabies and treated for tapeworm, to enter the UK without the need for a period of quarantine. This meant the removal for the legal requirement to treat dogs for ticks prior to entering the UK. As a consequence there are concerns that travelling dogs could return from continental Europe infested with ticks and/or infected with the diseases ticks carry [21, 22].

In February 2016, a cluster of cases of canine babesiosis were reported from the town of Harlow in Essex [23]. A number of dogs, examined at the same veterinary surgery, had no history of foreign travel. Subsequent testing of blood samples from dogs, ticks attached to the dogs and ticks collected from an area within Harlow where the dogs had been exercised, tested positive for *B. canis* indicating a potential source of the disease in dogs [24, 25]. The aims of this study are to report the testing conducted on the original outbreak of canine babesiosis in Harlow and a subsequent investigation of known *D. reticulatus* populations in England and Wales to assess the presence and distribution of *B. canis* infected *D. reticulatus* ticks. Sequence data obtained from this study suggested a single introduction of disease in this case rather than multiple introductions.

## Methods

### Field surveys

A tick survey was completed on 11th March 2016, in the town of Harlow, Essex, in the area where all infected dogs had been regularly exercised. The survey aimed to detect an active population of *D. reticulatus* within this area and to determine if questing ticks collected at the site were positive for *Babesia canis. Dermacentor reticulatus* populations from other locations across southern England such as Old Hall Marshes in Essex (collected in 2016) and Bolt Tail, located near Hope Cove in Devon (collected in 2011), as well as other *D. reticulatus* populations from different sites in Wales including Morfa Harlech (collected in 2010), Morfa Gwyllt near Tywyn (collected in 2012), and coastal grassland near Borth (collected in 2012) were included in this study and tested for the presence of *Babesia*.

All *D. reticulatus* specimens were collected by dragging a 1 × 1 m cloth over vegetation at the various locations across England and Wales. All ticks found attached to the cloth were collected, counted and stored in plastic vials during transit, frozen and stored at -80 °C. All ticks were identified morphologically using published keys [26, 27] and the species confirmed by sequence derived from the *cox*1 gene (see below).

### Detection of *Babesia* in thin blood smears

Samples of EDTA treated blood taken from the cephalic vein of affected dogs were used to prepare thin blood smears which were fixed in methanol, stained in 10% Gurrs improved R66 Giemsa [28] and examined under light microscopy at 1,000× magnification using oil immersion. A sample was considered positive if intraerythrocytic, single trophozoites and/or paired piriform merozoites of a large *Babesia* sp. were detected.

### Detection of piroplasms by polymerase chain reaction

For the dog blood samples and the *D. reticulatus* specimens from Essex, Devon and Wales, DNA extraction was carried out from EDTA-treated blood or manually homogenised tick leg, removed with haemolymph, using the DNeasy Blood and Tissue Kit (Qiagen, Manchester, UK), following the manufacturer’s instructions. DNA extractions were stored at 4 °C until tested by polymerase chain reaction (PCR). DNA extractions were performed from individual ticks. Samples were treated individually at each stage to reduce the risk of PCR contamination.

DNA samples were initially screened by real-time PCR using primers Bbmit2 and Bbmit3 (Table 1) that amplify an 150 base pair (bp) fragment of the cytochrome *c* oxidase subunit 1 gene (*cox*1), present within the mitochondrial genome. Subsequently, piroplasm genome was detected by real-time PCR using primers PIRO-A and PIRO-B (Table 1) that amplify a 423 bp piroplasm sequence [29]. The final PCR reaction mix for both PCRs (final volume 40 μl) consisted of: 13 μl H_2_O, 20 μl SYBR®Green JumpStart^TM^ Taq Ready Mix^TM^ (Sigma-Aldrich, Poole, UK), 1 μl of each primer (at 20 μM for primers PIRO-A and PIRO-B; 10 μM for primers Bbmit2 and Bbmit3) and 5 μl of the same DNA extract used above. Amplification was performed as follows: an initial denaturation step of 94 °C for 10 s followed by 45 cycles of 94 °C for 30 s, 58 °C for 30 s (when using the primers PIRO-A and PIRO-B; or 48 °C for 30 s when using primers Bbmit2 and Bbmit3), and 72 °C for 1 min. Samples were then sequenced using flanking primers at 1 pmol/μl using the ABI PRISM® BigDye® Terminator v3.1 Cycle Sequencing Kit (Applied Biosystems, Warringtom, UK), following the manufacturer’s protocols.

**Table 1:** Primer sequences used in this study

Gene	Gene	Sequence (5'–3')	Reference
Piroplasm 18S ribosomal RNA	Piro A Piro B	AATACCCAATCCTGACACAGGG TTAAATACGAATGCCCCCAAC	[29]
Piroplasm cytochrome *c* oxidase 1	Bbmit2 Bbmit3	CGACTTCTCTATTGTCTC ATGTCGTCTCACCATACC	This study
Tick cytochrome *c* oxidase 1	LCO1490 HCO2198	GGTCAACAAATCATAAAGATATTGG TAAACTTCAGGGTGACCAAAAAATCA	[30]

For the *D. reticulatus* specimens from Devon, ticks were pooled prior to RNA extraction (5 ticks/pool), which were screened by real-time PCR using primer PIRO-A and PIRO-B. The final PCR reaction mix (final volume 25 μl) consisted of: 6.25 μl H_2_O, 12.5 μl SYBR®Green JumpStart^TM^ Taq Ready Mix^TM^ (Sigma-Aldrich), 2 μl of each primer (at 20 μM primers PIRO-A and PIRO-B), 0.25 μl QuantiTect RT Enzyme (Qiagen) and 2 μl of RNA. Amplification was performed as described above.

### Molecular identification of ticks using *cox*1 sequence (DNA barcoding)

Polymerase chain reaction primers were those developed by Folmer et al. [30] (LCO1490 and HCO2198; Table 1), which are considered standard to amplify a 710-bp region of the *cox*1 gene. A reaction mix was prepared containing 2 μl of DNA template, 25 μl H_2_O, 5 μl of dNTPs (2 pmol/μl), 2.5 μl of MgCl_2_ (25 pmol/μl), 5 μl 10× buffer, 0.1 μl Bioline Taq Polymerase (Bioline Ltd), 5 μl of each primer (each at 10 pmol/μl), and 0.375 μl of Bovine Serum Albumin (20 mg/ml). The thermal profile consisted of: an initial denaturation step at 94 °C for 1 min, 5 cycles of pre-amplification of 94 °C for 1 min, 45 °C for 1.5 min, 72 °C for 1.5 min, followed by 35 cycles of amplification: 94 °C for 1 min, 57 °C for 1.5 min and 72 °C for one min, and a final elongation step of 72 °C for 5 min.

### Sequencing and sequence analysis

Piroplasm and tick amplicons generated by PCR were separated by electrophoresis in a 1% agarose gel impregnated with SYBR safe and visualised by UV illumination. Automated DNA sequencing using flanking primers was achieved as previously described [31]. Individual forward and reverse traces were oriented, edited, and aligned using the Sequencer (v.4.5; Genes Codes Corporation, Ann Harbour, MI, USA), GenDoc (v. 2.6.02) and ClustalX sequence analysis programs. Sequences were edited in Lasergene version 12.1 (DNASTAR) and assigned to a particular species, by BLAST (NCBI) search, when agreement was ≥ 98% to sequences of known taxa in GenBank (for *Babesia* spp.). In the case of tick *cox*1 barcode sequences, BLAST searches were performed at NCBI and BOLD databases for molecular species validation.

Once identification was achieved for *Babesia* spp., sequences were further analysed in MEGAv.6 [32]. Neighbor-joining (NJ) trees were exported as JPG files in Adobe Acrobat 8 Professional, and edited using Adobe Photoshop CS3 (v.10.0.1). The K2P distance metric was used to determine the intra- and inter-specific genetic distances, and a NJ tree analysis was carried out to represent the specimen’s clustering pattern. Bootstrap values were calculated to test the robustness of the cluster, which was obtained by conducting 1,000 pseudoreplicates.

## Results

**Fig. 1:**
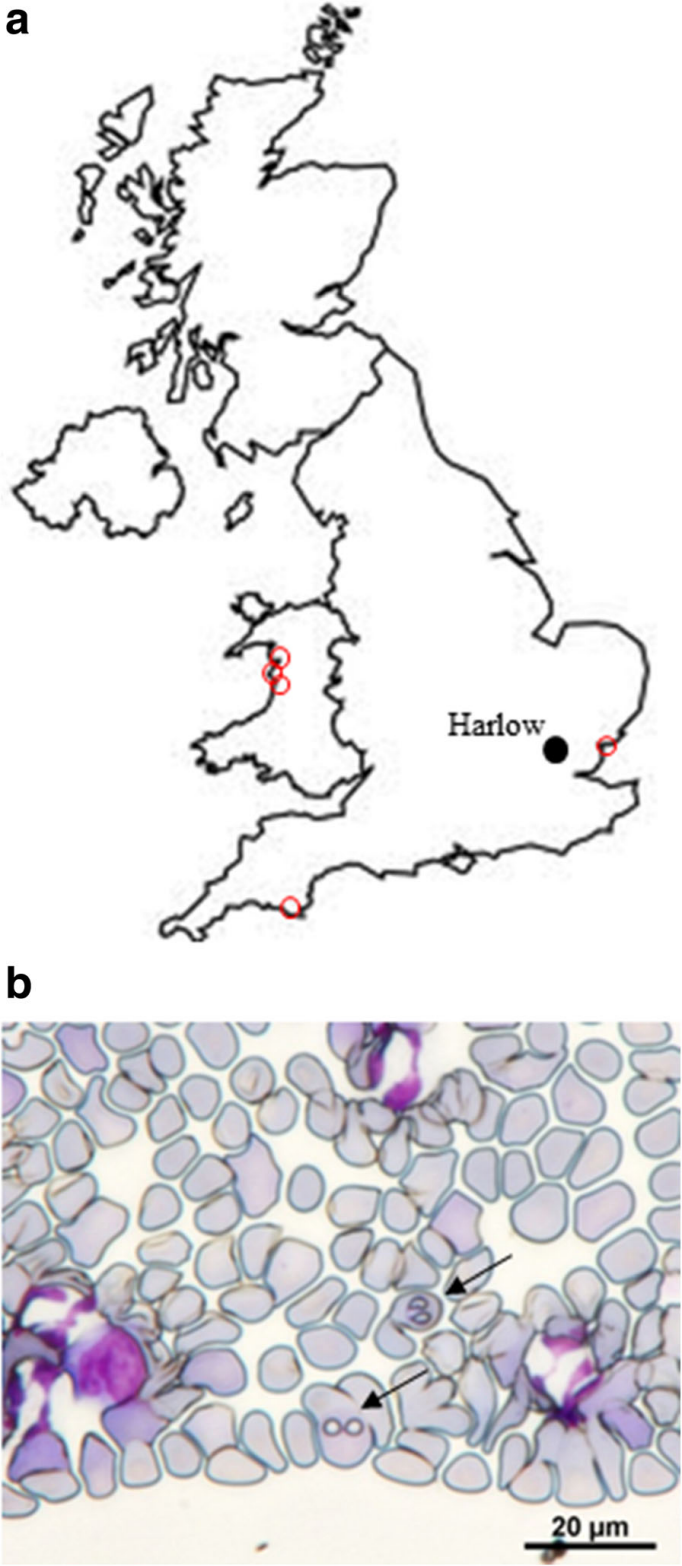
**a** Map of Great Britain showing the location of Harlow (closed circle) where all of the affected dogs were resident and the location of tick-collecting sites (open red circles). **b** Giemsa-stained blood smear from case 4 showing evidence of *Babesia* merozoites (arrows)

A summary of the clinical presentation of the dogs associated with the outbreak is provided in Table 2. The first dog presented with non-specific signs including lethargy on the 5th of October 2015 but babesiosis was not suspected at the time and only diagnosed retrospectively on a second visit the following year based on a positive PCR test. A second case presenting on the 26th of October 2015 raised suspicions of babesiosis, particularly as the dog had no history of travel outside the country, and a commercial test confirmed the presence of *Babesia* in a blood sample. The remaining dogs presented in the first three months of 2016. All of the dogs involved were resident in Harlow (Fig. 1a).

**Table 2:** Summary of clinical presentation and diagnostic investigation of affected dogs from Essex

Name (Case number)	Description	Date of original presentation	Clinical presentation	Blood work^a^	Diagnostic investigation by APHA^b^
Bud (1)	8.3 year old male Staffordshire Bull Terrier	5/10/2015	Dull, refusing food with pale mucous membranes	RBC: 4.55; Hb: 11.1; HCT: 32%	Blood smear negative; PCR positive
Mishka (2)	3.8 year old female Siberian Husky	26/10/2015	Dull, weak with pale mucous membranes. Confirmed *Babesia*-positive by commercial laboratory.	RBC: 2.11; Hb: 4.8; HCT: 12.5%	Not tested
Ruby (3)	9 year old female black Labrador	6/1/2016	Lethargic	RBC: 4.41; Hb: 11; HCT: 31%	Blood smear negative; PCR negative. A tick removed from the dog was positive by PCR
Bertie (4)	5.5 year old male Cavalier King Charles Spaniel	4/2/2016	Dull, listless with anorexia	RBC: 4.43; Hb: 11.6; HCT: 33%	Blood smear positive; PCR positive
Ollie (5)	10.6 year old male Cocker Spaniel	17/3/2016	Weak, dark urine, anorexia and pale mucous membranes	RBC: 4.1; Hb: 9; HCT: 27.7%	Blood smear positive; PCR positive. An engorged tick removed from the dog was positive by PCR

^a^Reference ranges: red blood cell count (RBC): 4.95–7.87; haemoglobin (Hb): 11.9–18.9, haematocrit (HCT): 36–55%

^b^Blood samples were tested by Giemsa staining of a blood smear or by pan–piroplasm PCR (see Methods section)

The first blood sample and an associated tick were received at APHA on the 8th February, 2016, and belonged to a dog which was found to be positive for *Babesia* by blood smear analysis by the attending veterinary surgeon and which was successfully treated for babesiosis. Both the tick removed from this dog and the blood sample, were tested for piroplasms using PCR and sequencing, which confirmed the presence of *Babesia canis* in the tick but not in the dog blood. Subsequent blood samples from additional dogs with disease suggestive of canine babesiosis were submitted for testing from Harlow. Of these, 3 of 5 blood samples were positive for *B. canis* either by blood-smear analysis (Fig. 1b) and/or the PCR, and the parasite was associated with either the blood-meal or the tick removed from four of the dogs affected. One engorged female tick was submitted live and maintained in a humid chamber for three weeks until oviposition. A leg taken from this tick and eggs derived from it were positive for *B. canis*.

**Fig. 2:**
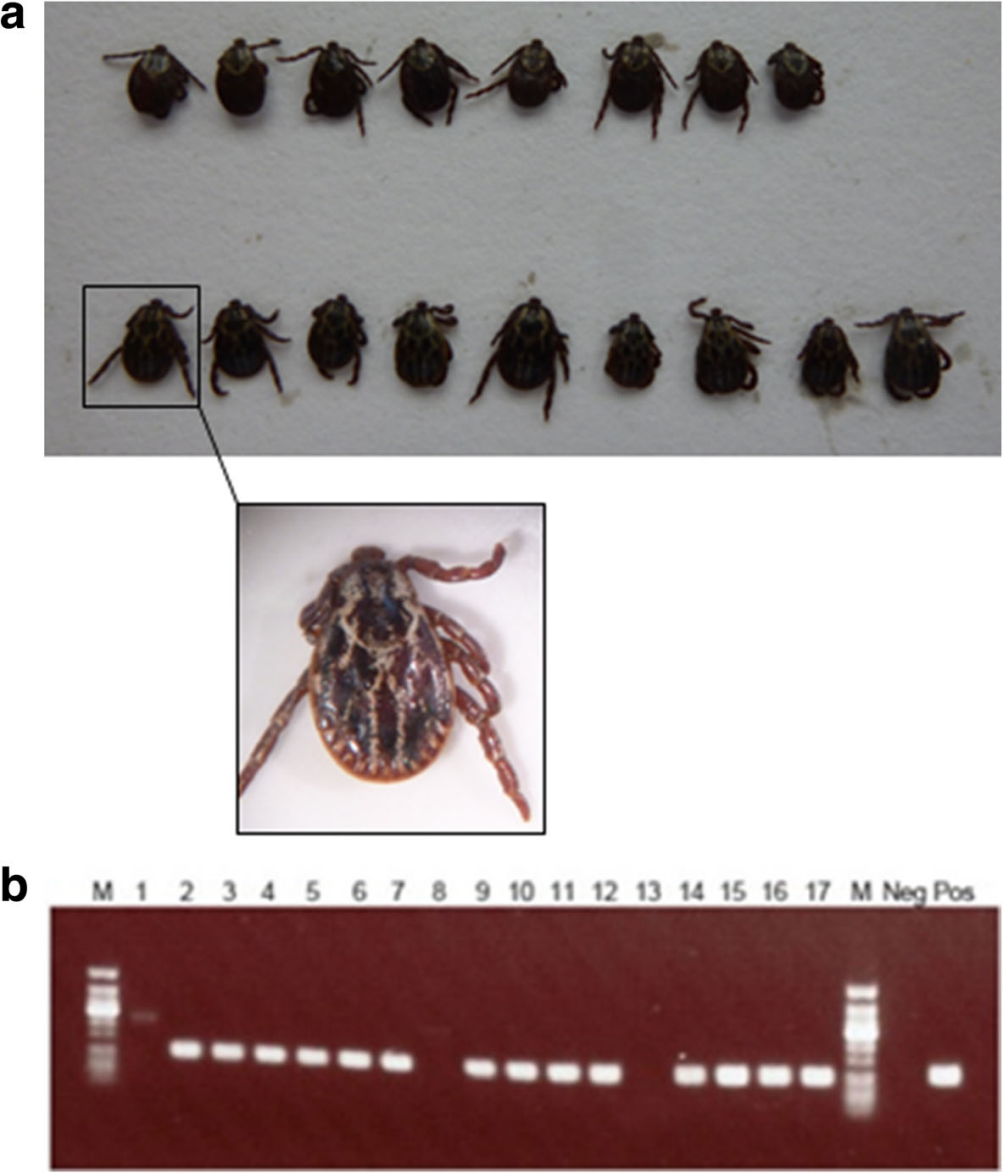
**a** Dorsal view of adult *D. reticulatus* ticks collected from a site in Harlow. Inset shows an enlarged image of the first male tick showing the distinctive ornamentation of the scutum. **b** Gel image showing *Babesia canis* mitochondrial amplicon products in *D. reticulatus* ticks collected in Harlow (Essex). The lane order is: 50 bp ladder (M), Female tick 1 (1), Female tick 2 (2), Female tick 3 (3), Female tick 4 (4), Female tick 5 (5), Female tick 6 (6), Female tick 7 (7), Female tick 8 (8), Male tick 1 (9), Male tick 2 (10), Male tick 3 (11), Male tick 4 (12), Male tick 5 (13), Male tick 6 (14), Male tick 7 (15), Male tick 8 (16), Male tick 9 n, 50 bp ladder (M), negative control (Neg), *Theileria annulata* as positive control (Pos)

**Fig. 3:**
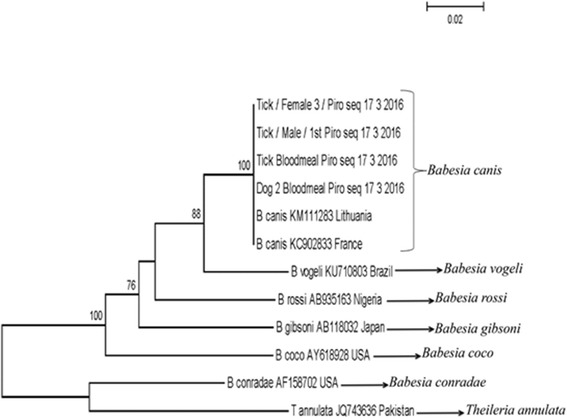
Neighbor-joining analysis of canine *Babesia* sequences derived from samples submitted from Harlow, Essex. A 364 bp fragment of the 18S rRNA was aligned with representative sequences obtained from GenBank. Bootstrap values higher than 80% are shown in the phylogenetic tree

The detection of a cluster of cases of canine babesiosis and associated *B. canis*-infected *D. reticulatus* ticks prompted a field survey of a site that most of the dogs had visited in the Harlow area. This survey collected 17 ticks (eight females, nine males; Fig. 2a) from one area close to a car park located next to Third Avenue and Abercrombie Way (51.762683, 0.088548). The area surveyed is a common dog-walking route, with dogs likely accessing this area as soon as they leave the car park with their owners as previously described by Hansford et al. [33]. The collected ticks were all identified as adult *D. reticulatus* based on morphology. Neighbor-joining (NJ) analysis using the *cox*1 gene sequence derived from each tick confirmed the morphological identification of the dog-associated and field collected ticks as *D. reticulatus* showing 100% identity to this species from continental Europe (data not shown). Pan-piroplasm PCR detected fourteen ticks (six females and eight males) positive for the presence of a piroplasm using assays that amplify either 18S sequence (data not shown) or a sequence of the mitochondrial genome (Fig. 2b). Sequencing of the amplicons derived from these samples established the presence of *B. canis* in these ticks. NJ analysis confirmed that the sequences derived from infected dogs, associated ticks and field-collected ticks were identical (Fig. 3), sharing 100% sequence identity with *B. canis* from Lithuania (GenBank KM111283) and France (GenBank KC902833). A representative sequence for this outbreak has been submitted to GenBank with accession number KY694436.

To further investigate the distribution of *B. canis* infection in UK ticks, a retrospective study of *D. reticulatus* collected between 2010 and 2016 were tested. DNA was extracted from ticks originating from southern England and Wales (Table 3). With the exception of the ticks sampled from Harlow, the majority of ticks tested (*n* = 95) were negative for piroplasm DNA (Table 3). However, one male *D. reticulatus* was found positive for the presence of piroplasm DNA from the population sampled from Harlech, Wales. Further sequencing analysis confirmed it was 100% identical to *Babesia canis* sequence available on GenBank (accession number KC207822) and to that found in samples from Harlow.

**Table 3:** Location and details of *D. reticulatus* tick collection in England and Wales (2010–2016)

Location	County	Year	Sample (All adults)	Piroplasm PCR result	*Babesia* species detected
Morfa Harlech	Wales	2010	3♂, 7♀	1/10	*Babesia canis*
Bolt Tail	Devon	2011	18♂, 37♀	0/55	–
Morfa Gwyllt near Tywyn	Wales	2012	2♂, 8♀	0/10	–
Borth	Wales	2012	3♂, 7♀	0/10	–
Old Hall Marshes	Essex	2016	3♂, 7♀	0/10	–
Harlow	Essex	2016	9♂, 8♀	14/17	*Babesia canis*

## Discussion

This study describes the first outbreak of *B. canis* in the UK with evidence for autochthonous transmission by indigenous ticks. There was no history of foreign travel by any of the dogs affected and ticks surveyed at a site visited by the dogs were positive for *B. canis*. A further case of *B. canis* infection has been reported from Essex [34]. This dog presented in November of 2015 with no history of travel outside of the county. The presence of the parasite in blood samples, ticks associated with the affected dogs and field collected ticks from a site known to have been visited by the infected dogs all point to Harlow, Essex as being the source of infection. The species of tick detected in the area, *D. reticulatus*, is a known vector of *B. canis* and has been associated with transmission of *B. canis* in northern Europe in Germany [35], the Netherlands [36] and Belgium [37]. In recent years, cases of canine babesiosis in the UK have been associated with travel to endemic countries, although the species of infecting *Babesia* is rarely reported. A single case of autochthonous transmission was suspected in a dog with no travel history [16] but no further investigations were conducted to support or refute this claim. Sequence analysis also suggested this case was more closely related to *B. vogeli*, than *B. canis*.

*Dermacentor reticulatus* is indigenous to the UK, although populations of this tick species are rare and prior to this study historical records suggested that this species was localised to a number of coastal sites [18]. A retrospective survey of *D. reticulatus* samples from known populations were tested for *Babesia* infection. Most populations tested were negative, but one location in Harlech, Wales, yielded a single positive specimen. No cases of canine babesiosis have been reported from this area [38] so the significance of this observation is unclear, and further surveys and testing would be required to assess the prevalence of infection in this tick population. Other pathogens have been detected in *D. reticulatus* from Wales [39].

The response to this outbreak illustrates a number of features. All of the cases detected were treated for babesiosis and recovered from infection. Information was disseminated rapidly through both the local, national and veterinary media outlets to raise awareness amongst dog owners and veterinary surgeons. Acaricide treatment was recommended as the current best practice guidance to prevent transmission while the importance of this for dogs returning to the UK from countries where canine babesiosis is indigenous was emphasised. Tick repellents could also be used to prevent tick infestation. Local measures to restrict access for dogs to the infested site were implemented by the local council and based on an understanding of vector behaviour, modification of the vegetation of the area was considered to reduce tick activity [40].

The source of infected ticks and canine babesiosis cases in Essex is presumed to be continental Europe. Although the mechanism for introduction remains speculative, it could have been introduced by an asymptomatically infected dog, as a recent arrival from abroad, providing a source of transmission to a tick (s) from a previously undetected population of *D. reticulatus* in the locality of the outbreak. Alternatively, a returning dog could have been infested with an infected tick (s) that dropped off at the site, causing the current infestation and subsequent outbreak. Either event is likely to have occurred in 2015 or earlier to produce an adult tick population that was *Babesia*-infected and sampled in 2016 [25]. Recent studies have shown that new populations of *D. reticulatus* are establishing in The Netherlands and Belgium, and that areas affected by this tick are increasing [41]. Alternative pathways for introduction include importation on livestock, horses or birds. Reports of autochthonous transmission of *B. canis* in previously disease-free areas have been reported from Norway [42] and Latvia [43].

## Conclusions

Currently, there is no evidence to confirm the route of introduction of *B. canis* into the UK in this case, but the underlying cause is likely to have been the movement of an infected or infested dog into the country. In addition to reducing the risk of further outbreaks of canine babesiosis, tick treatments that are effective and applied correctly, can considerably reduce the risk of introducing other non-endemic species. This could provide additional protection from the introduction of pathogens such as *B. caballi*, *Rickettsia conorii* and tick-borne encephalitis virus. Currently, acaricide treatment is recommended. The responsibility for tick control is entirely with pet owners. With over 100,000 dogs and cats entering the UK annually [20], the risk of further outbreaks of canine babesiosis caused by *B. canis* and the introduction of non-endemic ticks is heightened if owners do not treat their pets with an acaricide.

## Abbreviations

APHA: Animal and plant health agency
*cox*1: Cytochrome *c* oxidase subunit 1 gene
Hb: Haemoglobin
HCT: Haematocrit
PHE: Public health England
RBC: Red blood cell
